# MERTK Inhibition: Potential as a Treatment Strategy in EGFR Tyrosine Kinase Inhibitor-Resistant Non-Small Cell Lung Cancer

**DOI:** 10.3390/ph14020130

**Published:** 2021-02-06

**Authors:** Chao-Ju Chen, Yu-Peng Liu

**Affiliations:** 1 Department of Laboratory Medicine, Kaohsiung Medical University Hospital, Kaohsiung Medical University, Kaohsiung 807, Taiwan; chaoju.chen@gmail.com; 2 Graduate Institute of Clinical Medicine, Kaohsiung Medical University, Kaohsiung 807, Taiwan; 3 Research Center for Environmental Medicine, Kaohsiung Medical University, Kaohsiung 807, Taiwan

**Keywords:** EGFR-TKI resistance, non-small cell lung cancer, MERTK

## Abstract

Epidermal growth factor tyrosine kinase inhibitors (EGFR-TKIs) are currently the most effective treatment for non-small cell lung cancer (NSCLC) patients, who carry primary EGFR mutations. However, the patients eventually develop drug resistance to EGFR-TKIs after approximately one year. In addition to the acquisition of the EGFR T790M mutation, the activation of alternative receptor-mediated signaling pathways is a common mechanism for conferring the insensitivity of EGFR-TKI in NSCLC. Upregulation of the Mer receptor tyrosine kinase (MERTK), which is a member of the Tyro3-Axl-MERTK (TAM) family, is associated with a poor prognosis of many cancers. The binding of specific ligands, such as Gas6 and PROS1, to MERTK activates phosphoinositide 3-kinase (PI3K)/Akt and mitogen-activated protein kinase (MAPK) cascades, which are the signaling pathways shared by EGFR. Therefore, the inhibition of MERTK can be considered a new therapeutic strategy for overcoming the resistance of NSCLC to EGFR-targeted agents. Although several small molecules and monoclonal antibodies targeting the TAM family are being developed and have been described to enhance the chemosensitivity and converse the resistance of EGFR-TKI, few have specifically been developed as MERTK inhibitors. The further development and investigation of biomarkers which can accurately predict MERTK activity and the response to MERTK inhibitors and MERTK-specific drugs are vitally important for obtaining appropriate patient stratification and increased benefits in clinical applications.

## 1. Introduction

Lung cancer remains the leading cause of cancer-related death worldwide, and non-small cell lung cancer (NSCLC) represents the major histological subtype of the disease [1,2]. A subgroup of NSCLC patients, characterized by a high prevalence in never-smoker Asian females, respond well to epidermal growth factor tyrosine kinase inhibitors (EGFR-TKIs), due to the presence of sensitizing EGFR mutations, including the L858R point mutation in exon 21 and exon 19 deletions [3,4]. However, the long-term effectiveness of EGFR-TKIs is universally limited by the development of resistance. About 50% of EGFR-TKI-resistant patients can be explained by the acquisition of the EGFR T790M mutation, and the progression-free survival of these patients is a benefit of using osimertinib (AZD9291), which is a third-generation EGFR-TKI, as their second-line treatment [5]. The pathological mechanism for the other 50% of EGFR-TKI-resistant patients remains controversial. The overactivation of other pro-survival receptor tyrosine kinases (RTKs), such as HER2 and MNNG HOS transforming gene (MET) that share similar downstream signaling pathways with EGFR, is frequently observed in EGFR-TKI-resistant patients without the EGFR T790M mutation [6,7]. Many experimental settings demonstrate that targeting the “bypass” RTK-mediated signaling pathways is a potentially therapeutic strategy for overcoming the EGFR-TKI resistance (reviewed in ref. [8]).

However, the expression pattern and frequency of these receptors varies from patient to patient [9]. Thus, it is necessary to evaluate the therapeutic effect of targeting individual RTK in EGFR-TKI resistance.

**Figure 1:**
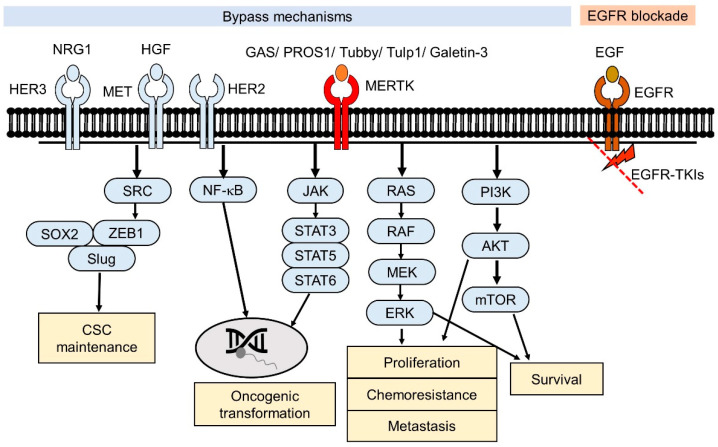
Effects of the Mer receptor tyrosine kinase (MERTK)-mediated signaling pathway in epidermal growth factor tyrosine kinase inhibitors (EGFR-TKI) resistance. For EGFR-TKI resistance without the acquired T790M mutation in EGFR, the downstream signaling pathways of EGFR are still blocked by EGFR-TKIs. Alternative RTK-mediated signaling pathways (bypass) contribute to the survival of the EGFR-TKI-resistant cancer cells. Through binding to their ligands, MERTK-induced signaling pathways may crosstalk with other RTK-mediated signaling pathways that promote cell survival.

The Mer receptor tyrosine kinase (MERTK), which is a member of the Tyro3-Axl-MERTK (TAM) RTK family, was initially cloned from a human B-lymphoblastoid expression library and predominantly expressed on monocytes, which are cells of epithelial and reproductive tissue [10]. MERTK is physiologically involved in natural killer-cell differentiation, the immune response, platelet aggregation, and the development of the central nervous system [11,12]. The dysregulation of MERTK has been linked to the pathology of many human diseases, such as atherosclerosis [13], retinitis pigmentosa [14], chronic obstructive pulmonary disease [15], thrombosis [16], acute liver failure [17], and diabetes [18]. In addition, MERTK contributes to the oncogenesis of a spectrum of human cancers, including hematological malignancies, glioblastoma, NSCLC, melanoma, breast cancer, colon cancer, gastric cancer, and prostate cancer [19]. Recently, evidence has showed that MERTK can function as a pro-angiogenic gene and promotes the metastasis of human cancers [20,21]. Several of these pathways were also observed to be involved in EGFR-downstream signaling, such as the activation of MAPK and PI3K/Akt cascades [8]. The collateral signaling events potentially causing the drug resistance in NSCLC are the presence of sensitizing EGFR mutations and ectopic MERTK expression (Figure 1).

TAM RTKs have been reported as novel anticancer targets through their promotion of tumor cell survival, proliferation, and migration [22,23]. Axl, which is the other member of the TAM family, has been implicated as a mechanism of resistance to EGFR-TKIs through bypassing inhibition of the original targeted oncogenic driver [8]. The DNA structure, biological ligands, and downstream signaling pathways are highly similar between Axl and MERTK [23]. Additionally, MERTK was revealed to be required for the surface accumulation of EGFR and downstream pathway activation [24]. Therefore, MERTK may represent a new target for therapeutic intervention in EGFR-TKI-resistant NSCLC.

In this review, we discuss the current understanding of mechanisms underlying EGFR-TKI resistance in NSCLC, the functions and regulation of MERTK, and the roles of MERTK in the resistance to EGFR-TKIs. We also present recent advances toward the introduction of small molecules targeting MERTK that have therapeutic potential.

## 2. Acquired EGFR-TKI Resistance in Lung Cancer

**Figure 2:**
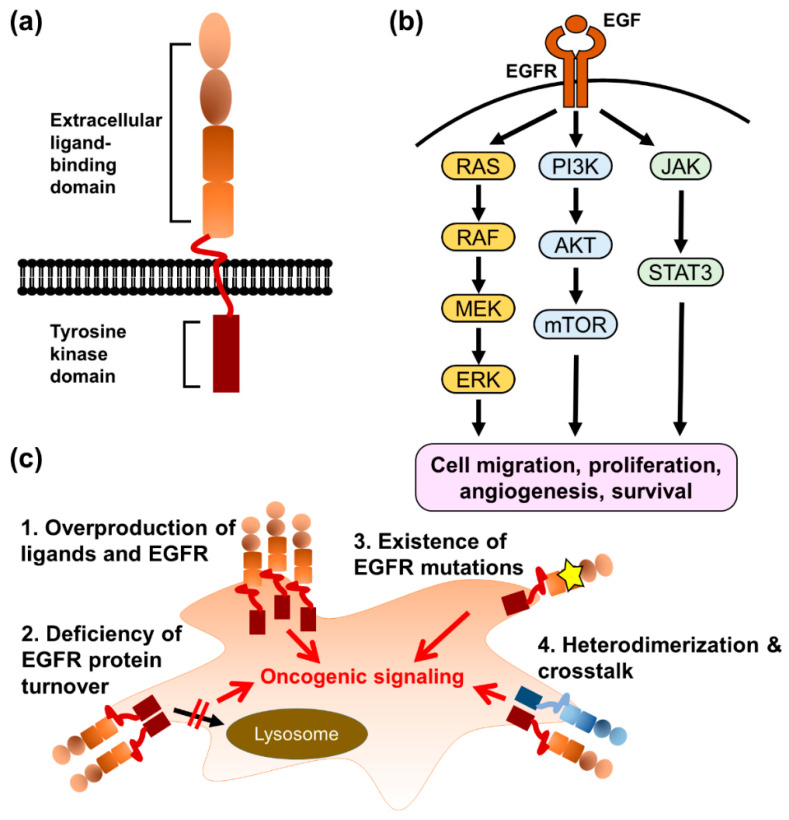
The mechanisms of acquired EGFR-TKI resistance. (**a**) The illustration of EGFR structure. (**b**) The downstream signaling pathways of EGFR contribute to cancer progression. (**c**) The *EGFR* gene amplification, deficiency of protein degradation, acquired T790M mutation, and different EGFR constitution are the major causes of EGFR-TKI resistance.

The epidermal growth factor receptor (EGFR/HER1/ERBB1) is a transmembrane protein consisting of an extracellular ligand-binding domain, a single transmembrane domain, and an intracellular domain, and is composed of a juxtamembrane segment, a kinase domain, and a C-terminal regulatory tail (Figure 2). EGFR is commonly expressed in epithelial, mesenchymal, and neuronal tissues and contributes to cell proliferation, differentiation, and development [25]. Several mouse models of EGFR deficiency have been established, but the phenotypes of EGFR deficiency are strain-dependent. Some EGFR-knockout mouse strains are embryonically lethal and other survival strains show developmental abnormalities in bonding, cardiovascular tissues, skin, and eyes [26,27,28]. In adults, the dysregulation of EGFR is involved in many pathological mechanisms of human diseases, such as renal fibrosis [29], coronary artery disease [30], autoimmune disease [31], chronic obstructive pulmonary disease [32], and cancers [33,34,35,36].

The binding of EGFR to its ligands, which are characterized by a consensus EGF motif containing six spatially conserved cysteine residues (CX7, CX4–5, CX10–13, and CXCX8) [37], induces the dimerization of two EGFR monomers. In the dimeric state, 12 of 20 tyrosine residues in the intracellular domain of each monomer are trans-phosphorylated and bind to downstream effectors, such as Shc1 and Grb2 [38], leading to the activation of several signaling pathways, including Ras/Raf/MAP kinase, PI3K/Akt, JAK/STAT, and phospholipase Cγ [39]. EGFR-mediated signaling pathways regulate various cellular functions in cancer cells, including cell migration, proliferation, angiogenesis, and survival [39]. The overactivation of EGFR and downstream signaling pathways in cancer cells can be triggered by different mechanisms, such as the overproduction of ligands and EGFR proteins; deficiency of EGFR protein turnover; existence of EGFR mutations, which cause the constitutive activation of EGFR; and cross-talk with alternative cell-surface receptors [40]. 

Epidermal growth factor receptor mutations are frequently observed in glioblastoma multiforme, breast cancer, and NSCLC [41,42,43,44]. The two most common EGFR-activating mutations in NSCLC are in-frame deletions in exon 19 and the L858R point mutation in exon 21, accounting for 45% and 40–45% of lung adenocarcinoma patients, respectively [4,7]. These mutations result in an asymmetric configuration of the kinase domain dimer, which leads to the ligand-independent activity of EGFR [45]. Cancer cells with the L858R mutations and exon 19 deletion may respond to the constitutive activation of the EGFR, which provides strong growth and survival signaling. Accordingly, targeting EGFR becomes a valuable strategy for the management of NSCLC patients who carry these active EGFR mutations.

Gefitinib, erlotinib, and afatinib are the EGFR-TKIs that reversibly (for gefitinib and erlotinib) or irreversibly (for afatinib) bind to the ATP-binding pocket in the kinase domain and block the kinase activity of EGFR. All of these EGFR-TKIs are recommended by international guidelines as a first-line treatment for NSCLC patients with sensitizing EGFR mutations [46]. A recent meta-analysis showed that these EGFR-TKIs had comparable effects on multiple pathological parameters of NSCLC patients receiving EGFR-TKIs as their first-line treatment [47]. Despite most of the patients responding well to the EGFR target therapy, tumor recurrence eventually occurs within 9–14 months in these patients, due to the development of drug resistance [48]. Several mechanistic themes underpinning the resistance to EGFR-targeted therapy have been studied [49]. 

The main mechanisms involved in the acquired resistance to EGFR-TKIs include secondary mutations in EGFR, phenotypic transformation, and the activation of alternative pathways [7]. The occurrence of a second EGFR mutation, called T790M, in exon 20 represents the most frequent mechanism and accounts for 60% of these cases [50]. Threonine 790 in the ATP-binding pocket of EGFR has been considered to be a “gatekeeper” residue. The T790M mutation increases the ATP affinity to the pocket domain of wild-type EGFR and the L858R mutant, thus reducing the potency of ATP-competitive EGFR-TKIs. To fight the acquired EGFR-TKI resistance, several small molecules targeting the EGFR T790M mutant have been discovered and some of these compounds have successfully entered different phases of clinical trials [51]. Osimertinib is the first third-generation EGFR-TKI that has been approved by The Food and Drug Administration (FDA) of the United States of America and The European Medicines Agency (EMA) of The European Union [52]. As a second-line treatment, osimertinb significantly prolonged the progression-free survival (PFS) of metastatic EGFR-mutant NSCLC patients who acquired the EGFR T790M mutation to 8.5 months compared to those receiving platinum therapy plus pemetrexed (PFS = 4.2 months) [53]. Interestingly, osimertinib also exhibited a higher therapeutic efficacy compared to that of standard EGFR-TKIs (gefitinib or erlotinib) in the first-line treatment of EGFR mutation-positive (with exon 19 deletion or the L858R mutation, but no T790M mutation) advanced NSCLC patients [54]. Unfortunately, the development of drug resistance to osimertinib, which may be due to acquired mutations other than T790M, remains a fundamental challenge for the disease control of NSCLC [55].

From the perspective of EGFR-TKI resistance without acquired EGFR mutations, even though the EGFR-TKIs efficiently block EGFR-mediated signaling pathways, the activation of other RTKs that drive compensatory downstream signaling pathways to EGFR may contribute to the growth and survival of cancer cells [56]. This hypothesis has been demonstrated by several pharmacological strategies using the combination of EGFR-TKIs with specific inhibitors targeting other RTKs [57]. Amplification of the genes encoding MET and epidermal growth factor receptor 2 (HER2/ERBB2) is frequently observed in recurrent tumors of NSCLC patients who received EGFR-TKIs as a first-line treatment [58]. There is an expanding spectrum of identified alternative signaling pathways which are activated by autocrine growth factor and cytokine signaling via cognate receptors, such as MET, BRAF, HER2, HER3, and TAM receptors [59].

## 3. Biological Function and Regulation of MERTK

**Figure 3:**
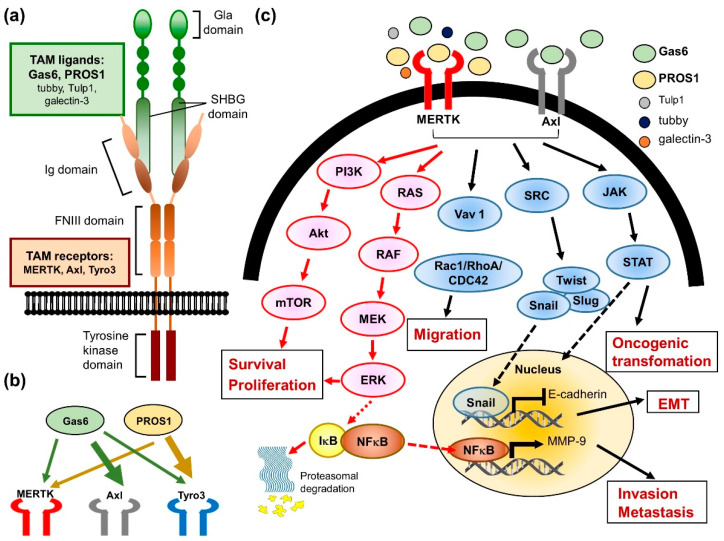
The biological role of MERTK in cancer cells. (**a**) The illustration of MERTK structure. (**b**) A model depicts the interaction of Tyro3-Axl-MERTK (TAM) receptors with their ligands (Gas6 and PROS1). The thickness of the arrows reflects the activation strength of each ligand to the TAM receptors. (**c**) Binding of ligands to the TAM receptors activates the downstream signaling pathways involving in cancer progression and drug resistance.

MERTK is a member of the TAM RTK family, which also includes Axl and Tyro3. This small family of RTKs are characterized by a combination of two immunoglobin-like domains and two fibronectin type III repeats in the extracellular region, a transmembrane portion, and a cytoplasmic kinase domain (reviewed in ref. [60]) (Figure 3a). In the early 1990s, each TAM receptor gene was first identified and cloned from a variety of species by independent groups. Thereafter, the TAMs were grouped into a unique RTK family in 1991 through PCR cloning their homology intracellular kinase domains [61]. The regulatory mechanism of TAM RTKs has been described in several progresses, including cell survival, proliferation, migration, adhesion, blood clot stabilization, the mediation of efferocytosis, and the modulation of inflammation (reviewed in ref. [60,62,63,64]). In normal adult tissues, MERTK, Axl, and Tyro3 display a widespread distribution pattern, with overlapping but unique expression profiles (Figure 3b). MERTK is expressed in hematopoietic cells, including monocytes, dendritic cells, natural killer cells, and platelets. High levels of MERTK expression are also found in the retina, kidney, lung, testis, and ovary. The heart, brain, and skeletal muscle also express lower levels of MERTK. Almost all adult tissue displays Axl expression. Axl is most abundantly expressed in the hippocampus and cerebellum, as well as monocytes, platelets, endothelial cells, the heart, skeletal muscle, the liver, the kidney, and the testis. Tyro3 is mainly expressed in the nervous system, and is also detected in hematopoietic cells, as well as the lung, kidney, and breast (reviewed in ref. [60]).

Unlike most of the RTKs that play essential roles in embryonic development, loss-of-function mutations in RTK genes often result in an embryonic-lethal phenotype. Even though the simultaneous genetic deletion of all three TAM RTKs is not embryonically lethal, it resulted in diverse phenotypes in a wide range of defects, such as a hyper-inflammatory state, multiple organ defects, and lymphoproliferation (reviewed in ref. [65,66]). The physiological regulatory roles of the TAM family are prominent in the innate immune control, reproductive, hematopoietic, hemostasis, vascular, and nervous systems. Disruption of the TAM receptor can lead to numerous diseases, including coagulopathy, autoimmune diseases, retinitis pigmentosa, chronic hepatitis, and neuron dysfunction (reviewed in ref. [64,65,66,67,68]).

The best-studied TAM RTK function is the role of MERTK in the macrophage-derived clearance of apoptotic cells, known as efferocytosis (reviewed in ref. [66]). The inability of macrophages from MERTK knockdown mice to efficiently clear apoptotic cells has been associated with the production of autoantibodies and development of autoimmune diseases, such as systemic lupus erythematosus [69,70]. The typical activation of RTKs requires ligand binding to the extracellular domain and induces receptor dimerization, subsequently leading to intermolecular autophosphorylation. In the progress of efferocytosis, the two best-characterized TAM ligands, which are growth arrest-specific protein 6 (Gas6) and vitamin K-dependent protein S (PROS1), may physically link TAM rece-ptors, generally MERTK or Axl, expressed on the surface of the phagocyte [71]. In addition, these ligands have two special structural characters that are key to their bioactivity. The first is a carboxy-terminally positioned sex hormone-binding globulin (SHBG) domain. (Figure 3a) This SHBG domain can bind to the Ig domains of the receptors and induce their dimerization and subsequent kinase activity. The other is the Gla domain, which is rich in glutamic acid residues. γ-carboxylation of the Gla domain allows it to bind to the lipid phosphatidylserine (PtdSer) with its amino terminus. PtdSer is abundant in the human body, but only available to activate TAM receptors when displayed on the extracellular membrane of apoptotic cells, exosomes, and aggregated platelets. With respect to hemostasis, the MERTK receptors are located on platelets and mediate thrombogenesis, platelet aggregation, and stabilization, and reduced thrombus formation was observed in Gas or MERTK knockout mice (reviewed in ref. [62,64,66]). Furthermore, three new MERTK ligands were identified more recently, including tubby, tubby-like protein 1 (Tulp1), and galectin-3, which were also shown to be involved in the MERTK-mediated phagocytosis and innate immune response [72,73].

Many studies have demonstrated that both MERTK and Axl are linked to survival, growth, and migration, although the role of Tyro3 is less well-defined (Figure 3c). Studies using chimeric MERTK receptors have elucidated that signaling pathways downstream of the MERTK kinases include growth factor-mediated proteins such as PI3K, Ras and MAPKs. These pro-survival signaling pathways may be activated in both normal and cancer cells. The aberrant regulation of these signaling pathways plays an important role in oncogenic transformation. Therefore, the overexpression of MERTK has been detected in numerous cancers, including myeloid and lymphoblastic leukemias, breast cancer, non-small cell lung cancer, brain cancers, melanoma, prostate cancer, liver cancer, colorectal cancer, gastric cancer, ovarian cancer, and rhabdomyosarcomas (reviewed in ref. [19,66]).

Proto-oncogenes can be activated by several mechanisms, such as gene mutations, amplification, and altered protein expression. To date, the overexpression of TAM family members, rather than mutation, appears to be the predominant effect with the development of cancer (reviewed in ref. [60]). MERTKs activate prosurvival signaling pathways, which was first shown in leukemia/lymphoma cells. In a transgenic model, the ectopic expression of MERTK in lymphocytes promotes the development of lymphoblastic leukemia/lymphoma. Stimulation of these cells with Gas6 induced MERTK autophosphorylation and the subsequent activation of extracellular signal regulated kinase (ERK1/2) and Akt [74]. In human T-cell lymphoblastic leukemia cell lines, the stimulation of MERTK by the ligand Gas6 led to activation of the prosurvival proteins ERK1/2 and MERTK-dependent activation of the STAT pathway and contributed to prosurvival phenotypes of tumor cells [75]. MERTK has been implicated in metastasis and linked to migration and invasion phenotypes through the regulation of FAK1, RhoA, Rac1, and CDC42 [61]. Furthermore, MERTK inhibition decreased colony formation, invasion, and xenograft growth in a human melanoma murine xenograft model [76]. Aside from its direct roles in tumor metastasis, MERTK may have additional functions in promoting metastasis through its role in negative regulation of the immune system (reviewed in ref. [77]). TAM RTK signaling in the suppression of natural killer cell activity has been demonstrated in promoted metastasis [78], while in MERTK knockout mice, it showed a pro-inflammatory state in the tumor microenvironment and resulted in the inhibition of tumor metastasis [79]. In addition, MERTK acts as an inhibitor of the innate inflammatory response to pathogens and balances the immune system. On the other hand, it may suppress antitumor immunity. Macrophages have long been recognized as an ancient cell type involved in host defense against pathogens. More recently, macrophages are being rediscovered as key regulators in the immunosuppressive tumor microenvironment [80,81]. Many studies suggest that tumor-associated macrophages correlate with a poor prognosis in patients with cancer [82,83,84]. Tumor-associated macrophages can produce immune-modulating and wound-healing cytokines, which may decrease the antitumor immunity and increase the growth of tumor cells [85]. A previous study showed that the loss of MERTK in the tumor microenvironment of MERTK knockout mice slowed the establishment, growth, and metastasis of mammary tumors. The results in this study indicated that MERTK in the tumor microenvironment aids malignant tumor progression by suppressing antitumor immunity [79].

## 4. The Role of MERTK in EGFR-TKI Resistance

**Figure 4:**
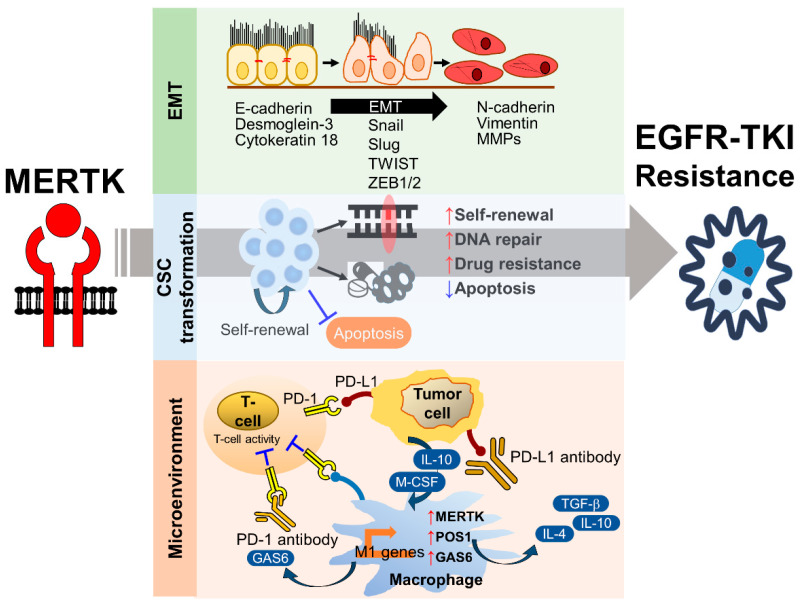
The mechanisms of MERTK-mediated EGFR-TKI resistance. The activation of MERTK contributes to epithelial-to-mesenchymal transition (EMT), cancer stem cell (CSC) maintenance, and the immunosuppressive microenvironment, which are the major biological factors involved in the development of EGFR-TKI resistance.

TAM-mediated signaling pathways are responsible for cell survival, proliferation, cell mobility, and tumor–host interactions, suggesting that the members of the TAM family may play important roles in the drug resistance of cancers. Multiple resistance mechanisms exist, including the intrinsic survival signaling in tumor cells, crosstalk of TAM RTKs as a bypass mechanism for other oncogene-targeted RTK inhibitors, and repression of the immune response in the tumor microenvironment (reviewed in ref. [22,66,86]). Much evidence strongly suggests that these receptors play major roles in resistance to conventional chemotherapy and targeted therapies (Figure 4).

The upregulation of MERTK can be observed in recurrent tumors of cancer patients who received target therapy or chemotherapy as their first-line treatment. In colorectal cancer, the upregulation of MERTK has been considered a predictive marker for resistance towards MEK1/2 inhibitors in a large clinical cohort [87]. The overexpression of MERTK was observed in melanoma cells with the BRAF V600E mutation, which are resistant to BRAF and MEK inhibitors [88]. In NSCLC, the excessive activation of Axl-mediated signaling pathways is associated with drug resistance to EGFR-targeted therapy [89,90,91]. However, the clinical relevance of MERTK and EGFR-TKI resistance is still unknown. A study using an in vitro cell culture showed that the overexpression of MERTK induced resistance to erlotinib in the PC9 cell line, which is an EGFR-TKI-sensitive cell line [92]. Furthermore, a specific MERTK inhibitor, UNC2025, induced apoptosis of NSCLC cell lines carrying the EGFR T790M mutation, leading to EGFR-TKI resistance [93]. In addition to its role in EGFR-TKI resistance, MERTK may also contribute to chemoresistance in NSCLC. The knockdown of MERTK by short hairpin RNAs (shRNAs) improved the sensitivity to chemotherapeutic agents by promoting apoptosis [94]. A monoclonal antibody targeting MERTK increased the chemosensitivity of NSCLC cells to carboplatin [95].

The contribution of the ligand-dependent canonical RTK signaling pathway of MERTK and Axl in drug resistance has been extensively explored [96]. One study demonstrated that the post-translational processing of membrane-bound Axl by γ-secretase-mediated proteolysis contributed to the nuclear localization of the cleaved intracellular domain of Axl [97]. Although the biological function of the Axl intracellular domain in the nucleus is unknown, Axl with specific mutations between amino acid 452 and 472 resisted γ-secretase and contributed to the chemotherapy of NSCLC cell lines.

Importantly, the signaling cascades activated downstream of Axl/MER are dependent on the context in which the ligands and microenvironment are presented to the TAM extracellular domains. A previous study observed that TAMs have distinct and dynamic patterns of activation by Gas6 and PROS1, representing the two best characterized TAM ligands. Gas6 binds to both MERTK and Axl, while PROS1 only binds to MERTK [96,98]. Furthermore, in the non-inflammatory microenvironment, MERTK shows weak activation toward both ligands and the affinity of Gas6 for Axl is significantly stronger than its affinity for MERTK. In contrast, in the presence of PtdSer lipid vesicle and apoptotic cells, Gas6 changes the strong binding affinity from Axl to MERTK [66,96].

The upregulation of MERTK has been observed in many cancers. Because MERTK and EGFR share PI3K/Akt and MAPK as their downstream signaling pathways, the targeting of MERTK by small molecules or monoclonal antibodies becomes a potential strategy for overcoming EGFR-TKI resistance in NSCLC. A recent study showed that the ectopic expression of MERTK induced erlotinib resistance in PC9 cells, which carry an exon19 deletion of EGFR, inducing drug resistance to erlotinib treatment [92]. It has been shown that MERTK is involved in the phenotypic and genotypic alterations of cancer cells and microenvironmental regulation. Here, the roles of MERTK in EMT, CSC maintenance, and the immunosuppressive microenvironment are discussed.

### 4.1. The Role of MERTK in EMT-Associated EGFR-TKI Resistance

Epithelial-to-mesenchymal transition is a biological process where epithelial cells morphologically and physiologically convert into a mesenchymal state, which is characterized by a loss of cellular polarity, reduced expression of junctional proteins (e.g., E-cadherin, Desmoglein-3, and Cytokeratin 18), greater motility and invasiveness, and the gain of mesenchymal markers (e.g., Vimentin and Matrix metalloproteinases). In addition to its roles in embryonic development and tissue repairing, EMT has been associated with metastasis, drug resistance, and stemness in multiple cancers. The upregulation of EMT inducers and downregulation of EMT inhibitors are frequently found in patients with an acquired drug resistance to chemotherapeutic agents and targeted therapies [99,100]. In the past decades, a number of intrinsic EMT inducers have been identified, including transcription factors (e.g., Snail, Slug, TWIST, and ZEB1/2), microRNAs (e.g., miR-200c and miR-34), polycomb group proteins (e.g., Bmi1 and EZH2), and long non-coding RNAs (e.g., lncRNA Val) [101]. Furthermore, many extracellular stimuli, including inflammation, hypoxia, DNA damage agents, and the extracellular matrix, are associated with EMT and tumor progression. Although the molecular mechanism of EMT-related EGFR-TKI resistance is largely unknown, reversed EMT by an ectopic expression of E-cadherin or a long-term withdrawal of EGFR-TKI sensitizes the non-T790M EGFR-TKI-resistant NSCLC cell lines to the EGFR-TKI treatment [102,103].

#### 4.1.1. TGF-β-Induced EMT and EGFR-TKI Resistance

EMT can be triggered by a number of growth factor- and mitogen-mediated signaling pathways, which are mostly activated by RTKs. These EMT-related signaling pathways not only contribute to migration and invasion, but also promote proliferation, survival, and differentiation. Transforming growth factor β (TGF-β) is a potent EMT inducer. The binding of TGF-β to its receptors activates the canonic Smad2/3/4 signaling pathway that represses E-cadherin expression and activates Snail, Slug, and ZEB1/2 expression. Although the TGF-β receptor is a serine-threonine kinase, the phosphorylation of tyrosine residues in its cytoplasmic domain induces the activation of non-canonic PI3K/Akt/mTOR and Ras/Raf/MAP kinase pathways, which promote cell survival and proliferation. Transient exposure to TGF-β induces EMT and acquired EGFR-TKI resistance in NSCLC cell lines. TGF-β/Smad-induced EMT contributes to Axl-mediated gefitinib resistance in PC9 cells [104]. The activation of Axl or MERTK by Gas6 inhibits TGF-β-induced EMT [105]. 

#### 4.1.2. Hippo Pathway-Mediated EMT and EGFR-TKI Resistance

**Figure 5:**
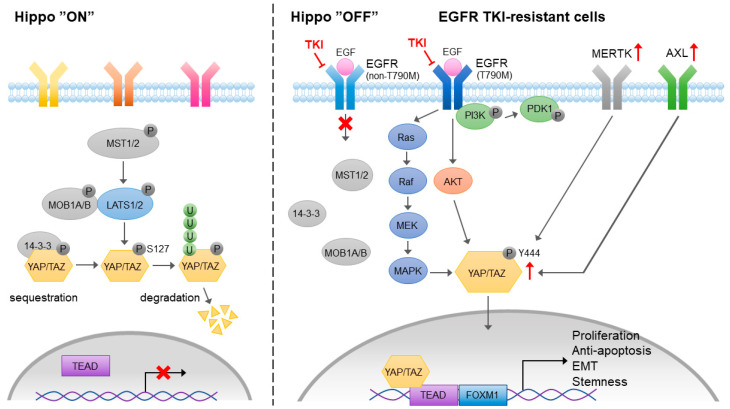
The role of MERTK in Yes-associated protein (YAP)-mediated EGFR-TKI resistance. Activation of hippo pathway blocks the nuclear translocation of YAP or promotes YAP degradation. In the EGFR-TKI resistant cancer cells, MERTK and Axl are upregulated. EGFR-mediated PI3K-independent Akt and MAPK signaling pathways contribute to the activation of YAP. In addition, MERTK may directly interact with YAP and phosphorylate YAP at Y^444^ residue, which promotes its nuclear localization and transcriptional activity.

The Hippo pathway is an evolutionarily conserved pathway that controls organ development, tissue regeneration, cell growth, and differentiation. In cancer, the Hippo pathway is involved in tumor growth, survival, migration, and stemness. In mammalian cells, the Yes-associated protein (YAP) and its paralog transcriptional coactivator with a PDZ-binding motif (TAZ) are the key transcription coactivators that translocate to the nucleus and interact with TEA domain family member (TEAD) transcription factors, leading to the activation of downstream genes that are involved in proliferation, anti-apoptosis, migration, and stemness maintenance (Figure 5). The activation of YAP/TAZ/TEAD is negatively regulated by the Hippo signal cascade. The auto-phosphorylation of MST kinases, including the MST1 and MST2 heterodimer, phosphorylates large tumor suppressor 1 and 2 kinases (LATS1 and LATS2) and MOB kinase activator 1A and 1B (MOB1A and MOB1B) and forms an MOB/LATS complex. The interaction of phosphorylated MOB1A/MOB1B with LATS1/LATS2 also leads to the auto-phosphorylation of LATS1 and LATS2 at Ser909 and Ser872, respectively. YAP and TAZ are the substrates of activated LATS1/LATS2. The phosphorylation of YAP/TAZ by LATS1/LATS2 results in their cytoplasmic sequestration through binding to 14-3-3 proteins or protein degradation by ubiquitination. 

Although YAP has no DNA binding activity, it interacts with EMT-associated transcription factors that suppress the expression of epithelial markers and promote tumor progression [106]. The induction of YAP-mediated EMT contributes to proliferation, migration, invasion, and drug resistance in lung cancer [107,108]. It has been shown that YAP is upregulated in EGFR-TKI-resistant lung adenocarcinoma [109], which may be mediated by an Akt and MAPK-independent PI3K/PDK1 pathway [110]. An experimental setting showed that targeting YAP reversed EGFR-TKI resistance in NSCLC cells [111]. Combined treatment of EGFR-TKI and a YAP inhibitor prolonged survival among lung cancer patients [109]. Although the mechanism of YAP-mediated EGFR-TKI resistance is not clear, the activation of YAP is capable of promoting EMT, leading to the induction of EGFR-TKI resistance in lung cancer [112]. A recent study showed that the EMT-associated EGFR-TKI resistance was mediated by a YAP/FOXM1 axis [108].

In addition to its upstream regulators in Hippo pathways, YAP/TAZ has been shown to crosstalk with a variety of receptor-mediated signaling pathways, such as G-protein-coupled receptor, Notch, TGF-β, Wnt, Integrin, EGF, and VEGF signaling pathways. The activation of EGFR inactivates the upstream negative regulators of YAP through PI3K and PDK1-independent Akt phosphorylation. EGFR is also known to activate YAP through the Ras/Raf/MAPK signaling cascade. Upon the development of EGFR-TKI resistance, both Axl/MERTK and YAP are upregulated. It has been shown that MERTK directly interacts with and phosphorylates YAP, which is independent of upstream Hippo signaling, and contributes to tumorigenesis [113]. Gas6-mediated activation of Axl induces EMT and YAP expression, leading to EGFR-TKI resistance [114]. The results from these studies indicate that the Axl/MERTK signaling pathway is one of the positive upstream regulators of YAP that contributes to EMT-associated EGFR-TKI resistance. Interestingly, Axl may also be the downstream target of YAP that sustains YAP-driven EGFR-TKI resistance in EGFR-addicted lung cancer cell lines [115]. Both Axl/MERTK and the Hippo signaling pathway contribute to the acquired EGFR-TKI resistance, and positive feedback between Axl/MERTK expression and YAP activity may strongly sustain the EGFR-TKI resistance.

### 4.2. The Role of MERTK in the Cancer Stem Cell Phenotype

Most solid tumors contain a rare population of tumor cells that harbor stem cell properties, such as self-renewal, differentiation, and drug resistance. Similar to their corresponding tissue stem cells, CSCs have greater DNA repair, anti-oxidative stress, anti-apoptosis abilities compared to differentiated cancer cells. In addition, CSCs express higher ABC transporters that can eliminate anti-cancer agents from the cells. In solid tumors, most of the CSCs are in a quiescent state, which protect them from the attack of DNA damage drugs and allows timely DNA repair. The origin of CSCs in tumors is still debated. The clonal evolution hypothesis depicts that the accumulation of oncogenic mutations contributes to the transformation of normal stem cells to CSCs. On the other hand, differentiated cancer cells may acquire CSC phenotypes by exposure to microenvironmental stress, such as inflammation, oxidative stress, and DNA damage. Recently, the CSC phenotype has been linked to acquired EGFR-TKI resistance [116]. Stemness-associated genes are upregulated in the tumors of EGFR-TKI-resistant NSCLC patients compared to their expression levels in the tumors of EGFR-TKI-sensitive patients [117]. Both stemness- and senescence-associated proteins are upregulated in gefitinib-tolerated lung adenocarcinoma cell lines [118]. The treatment of EGFR-TKIs induces YAP-mediated anti-apoptosis and dormancy of cancer cells, representing a phenotype of CSCs [119]. The activation of stemness-associated signaling pathways contributes to the acquired EGFR-TKI resistance [120]. Pharmacological and genetic approaches targeting CSC phenotypes effectively increase the drug sensitivity of EGFR-TKI-resistant NSCLC to the treatment of EGFR-TKIs [121,122].

Although it is still unknown how the CSC phenotype is provoked during the acquisition of EGFR-TKI resistance, several possible mechanisms have been proposed. It has been shown that the downregulation of E-cadherin generated the CSC phenotype and drug resistance to osimertinib in NSCLC cell lines [123]. The inhibition of NF-κB activity by Withaferin A significantly depleted CD133-positive CSCs in gefitinib-resistant lung adenocarcinoma cell lines [118]. Several histone-modifying enzymes and pro-survival signaling pathways, e.g., STAT3 and Akt/mTOR, are also involved in the acquired CSC phenotype and EGFR-TKI resistance [124,125]. So far, research on the role of MERTK in lung CSCs is lacking. It has been shown that MERTK was upregulated in glioblastoma multiforme (GBM) [126]. The upregulation of MERTK is associated with an increase in invasion, the CSC phenotype, and resistance to DNA damage-induced apoptosis in glioma cells. This MERTK-mediated CSC maintenance may be controlled by a STAT3-kRas/Src-signaling cascade [127]. In prostate cancer, the activation of TAM receptors in disseminated prostate cancer cells by the osteoblast-derived Gas6 in the bone marrow induces G1 arrest and an increase in the CSC population in the microenvironment of bone marrow [128]. However, the same research group reported a further study demonstrating that the shRNA-mediated MERTK knockdown, but not Axl and Tyro3, resulted in the upregulation of stemness-associated genes, anti-apoptosis, G1-G0 arrest, and the characteristics of dormancy in prostate cancer cells [129]. These findings suggest that the function and activity of MERTK in CSC maintenance can be regulated by microenvironmental factors. In addition, although MERTK, Axl, and Tyro3 share the same ligands, MERTK may activate specific signaling pathways that elicit unique cellular behaviors independent of Axl and Tyro3. In lung cancer, the CSC phenotype can be induced by the signaling pathways that also crosstalk with MERTK-mediated signaling pathways, e.g., STAT3, PI3K/Akt, and Ras/Raf/MAPK. Further study is required to investigate whether the activation of MERTK may induce the CSC phenotype and lead to EGFR-TKI resistance as a consequence.

### 4.3. The Role of MERTK in Microenvironment Remodeling

**Figure 6:**
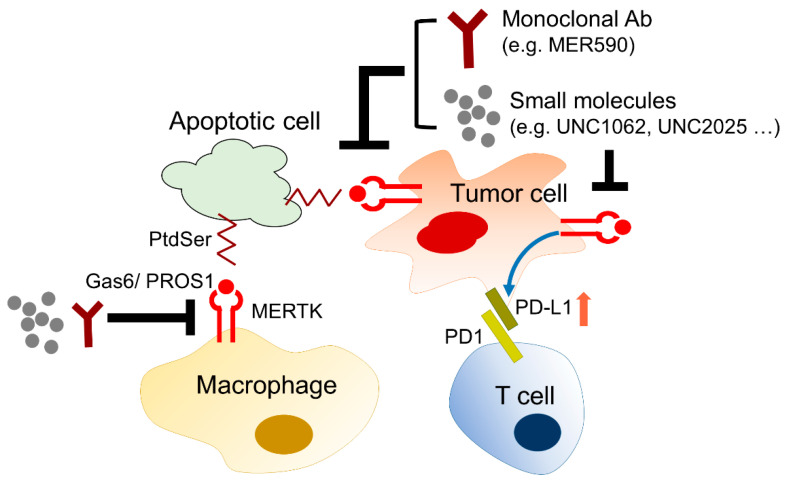
The role of MERTK in microenvironment remodeling. MERTK expressed in cancer cells promotes cell proliferation and survival. In the tumor microenvironment, MERTK-mediated signaling pathways enhance PD-L1 expression in cancer cells leading to the immunosuppression of CD8+ T cells. In addition, apoptotic cancer cells can be eliminated by macrophages through efferocytosis via a PtdSer-Gas6-MERTK, so called “eat me”, signal. Inhibition of MERTK activity by small molecules or monoclonal antibodies may enhance the therapeutic efficacy of anti-cancer agents.

The biological and chemical interactions between tumor cells and the microenvironment critically contribute to all hallmarks of cancer, including angiogenesis, inflammation, metabolic alterations, immune suppression, metastasis, and therapeutic resistance. The stromal cells, such as tumor-associated macrophages, neutrophils, tumor-infiltrating lymphocytes, tumor-associated fibroblasts, dendritic cells, and endothelial cells, influence tumor progression and the therapeutic efficacy by directly interacting with tumor cells, secreting growth factors and cytokines, constructing the extracellular matrix, and metabolizing drugs and stress factors. MERTK is not only upregulated in tumor cells, but also excessively expressed in the surrounding monocytes, macrophages, dendritic cells, and T lymphocytes. Activation of the MERTK signaling pathway induces an immune-suppressive microenvironment via regulating inflammatory cytokine-mediated signaling pathways, the PD-1 and PD-L1 axis, and efferocytosis of macrophages (Figure 6).

#### 4.3.1. The Role of MERTK in Immune Checkpoint Inhibitor Therapy

Programmed cell death protein-1 (PD-1), which is an immune checkpoint receptor, is expressed in lymphocytes and myeloid cells. After binding to its ligands, programmed death-ligand 1 (PD-L1), which is broadly expressed in healthy tissues and tumor cells, negatively regulates the effector functions of T cells, and promotes T-cell tolerance and escape from host immunity by downregulating CD8+ T-cell survival. Accordingly, immune checkpoint inhibitors targeting the PD-1/PD-L1 axis have been applied as an anti-cancer therapy in many cancer types, including NSCLC. Currently, four monoclonal antibodies targeting PD-1 (pembrolizumab and nivolumab) and PD-L1 (atezolizumab and durvalumab) have been approved by the U.S. Food and Drug Administration (FDA) for the treatment of advanced NSCLC. However, patients with EGFR-mutant advanced NSCLC were limited in or excluded from the clinical trials of the four immune checkpoint inhibitors [130,131,132,133]. So far, a clinical trial investigating the therapeutic effect of immune checkpoint inhibitors in EGFR-TKI-resistant NSCLC patients is lacking. More studies are required to examine the regulation of the PD-1/PD-L1 interaction and immune microenvironment in EGFR-resistant tumors.

##### The Regulation of PD-L1 Expression by MERTK

PD-L1 is frequently upregulated in many cancer cells, including NSCLC cells. The high levels of PD-L1 expression can be explained by the activation of oncogenic signaling pathways, such as PI3K/Akt/mTOR and Ras/Raf/MAPKs, which are also the downstream signaling pathways of EGFR and MERTK [134,135]. The correlation between the EGFR status, EGFR-TKI resistance, and PD-1/PD-L1 expression has recently been revealed in NSCLC. In NSCLC patients who received gefitinib as their first-line treatment, the PD-L1 expression in tumor cells was markedly increased after gefitinib treatment [136]. In addition, the induction of PD-L1 expression by acquired EGFR-TKI resistance contributes to the immune escape of NSCLC cells [137]. These findings suggest that the mechanisms contributing to EGFR-TKI resistance may also trigger PD-L1 expression and promote the immune escape of tumor cells. It has been shown that the activation of MERTK-mediated signaling pathways through binding to Gas6 or ectopic expression of a constitutively activated MERTK significantly induces PD-L1 mRNA and protein expression [138]. In contrast, the reduction of MERTK and its downstream signaling pathway by shRNA-mediated knockdown, treatment of the MERTK inhibitor, BMS-777607, or the PI3K inhibitor decreases PD-L1 expression. In addition to the regulation of PD-L1 expression in cancer cells, the expression of PD-L1 in monocytes and macrophages was downregulated in MERTK-deficient mice or in MERTK-wild-type mice which were treated with an MERTK inhibitor called MRX-2843 [139]. Although MERTK is not dominantly expressed in T cells, the inhibition of MERTK indirectly upregulates PD-1 expression in CD4+ and CD8+ T cells, leading to T cell activation [139]. These findings suggest that targeting MERTK may enhance the therapeutic effect of immune checkpoint inhibitors. So far, there has been no study or clinical trial evaluating the therapeutic effect of the combination of MERTK inhibitors and anti-PD-1/PD-L1 agents in NSCLC, especially in patients with TKI-sensitive EGFR mutations. However, in a recent animal study of triple-negative breast cancer, the combination of the MERTK inhibitor BMS-777607 and an anti-PD-1 monoclonal antibody synergistically decreased tumor growth and the incidence of lung metastasis [140]. Since the excessive activation of MERTK-mediated signaling pathways is involved in EGFR-TKI resistance in NSCLC, the combination of MERTK inhibitors and anti-PD-1/PD-L1 agents may shed light on overcoming EGFR-TKI resistance in NSCLC.

##### The Regulation of MERTK by PD-L1-Mediated EGFR-TKI Resistance

In addition to its immune-suppressive role in cancer progression, the direct function of PD-L1 in tumor cells has been neglected thus far. A cohort study showed that high PD-L1 expression (>50% of immunohistochemistry reactivity) in treatment-naïve tumors predicts a poor EGFR-TKI response and prognosis of NSCLC patients with TKI-sensitive mutations [141], suggesting that PD-L1 may contribute to EGFR-TKI resistance in NSCLC. Although the PD-L1 protein lacks enzyme activity, the intracellular domain of PD-L1 contains three evolutionarily conserved motifs, known as the RMLDVEKC, DTSSK, and QFEET motifs, which can interact with some signaling molecules that are involved in the activation of pro-survival signaling pathways or the inhibition of some pro-apoptotic signaling pathways [142]. One study showed that the overexpression of PD-L1 led to YAP-mediated EGFR-TKI resistance in NSCLC cells, although the detailed molecular mechanism is not clear [143]. In addition, the ectopic expression of PD-L1 promotes TGF-β-mediated EMT and EGFR-TKI resistance in NSCLC cells through upregulating Smad3 phosphorylation [144]. Interestingly, PD-L1 may translocate into the nucleus and interact with Sp1, which promotes Gas6 expression and secretion, leading to activation of the MERTK signaling pathway and the proliferation of NSCLC cells [145]. Although direct evidence of the PD-L1-regulated MERTK signaling pathway is lacking, a study of the PD-L1 signalosome showed that the intracellular domain of PD-L1 may interact with molecules that are involved in Akt/mTOR, the DNA damage response, and the anti-apoptosis pathway [142,146]. Since the activation of MERTK is one of the EGFR-TKI-resistant mechanisms, especially to the non-T790M EGFR mutation, the PD-L1-induced MERTK signaling pathway may explain the PD-L1-mediated EGFR-TKI resistance.

#### 4.3.2. The Role of MERTK in Efferocytosis

**Figure 7:**
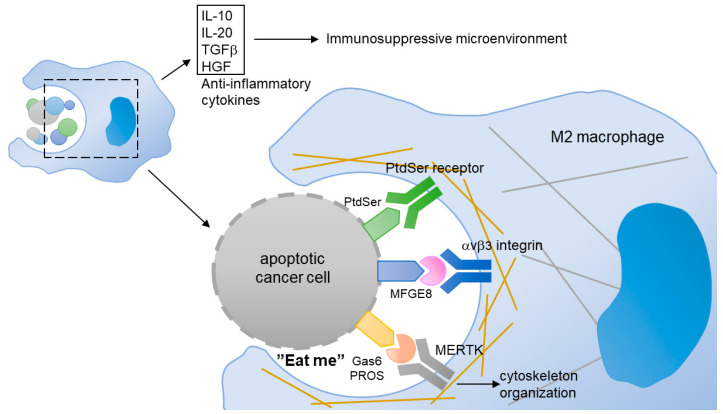
In tumor, M2-like macrophages scavenge the apoptotic cancer cells through a process of efferocytosis. Gas6 or PROS binds to the PtdSer presented on the membrane of apoptotic cells. The MERTK expressed in M2-like macrophages interacts with the Gas6/PtdSer complex, and induces cytoskeleton reorganization. In addition, MERTK expressed in macrophage can contribute to the release of anti-inflammatory cytokines that build an immunosuppressive microenvironment.

Phagocytosis is a cellular process for removing infectious microbes, foreign substances, and apoptotic cells by macrophages. In cancer, monocytes and macrophages are recruited to the tumor microenvironment, through a process guided by the tumor or stromal cell-derived chemokines (Figure 7). The infiltrated monocytes differentiate into macrophages and acquire a specific tumor-associated macrophage phenotype. Most of the tumor-associated macrophages resemble M2-like macrophages. Unlike M1-like macrophages with pro-inflammatory and anti-cancer activities, the M2-like macrophages function as immunosuppressive cells contributing to tumor initiation, angiogenesis, drug resistance, and metastasis [147,148]. The M2 macrophages help the immunosuppressive microenvironment by releasing anti-inflammatory cytokines, such as IL-20, TGF-β, and HGF [149], or by preventing the release of intracellular antigens from apoptotic cancer cells via efferocytosis [150]. 

MERTK is involved in the innate immune response by regulating the efferocytosis of macrophages. Although MERTK is not expressed in circulating monocytes, it is upregulated during the transformation of monocytes into tumor-associated macrophages, especially M2 macrophages [151]. The PtdSer, which is exposed on the apoptotic cancer cells, binds to PROS and Gas6 and transmits an “eat me” signal to the macrophages, which express abundant MERTK [13]. The upregulation of MERTK in M2 macrophages enhances their efferocytosis [152]. As such, the activation of MERTK increased the production of anti-inflammatory cytokines [151]. It is worth noting that Gas6 is produced and released from cancer-associated fibroblasts and M2 macrophages, suggesting a positive feedback loop that drastically reinforces the immunosuppressive environment in tumors [153]. 

Since M2 macrophages contribute to maintenance of the tumor immunosuppressive microenvironment that promotes drug resistance and immune escape through activation of the MERTK signaling pathway, targeting MERTK has become a potential therapeutic strategy for fighting NSCLC. It has been shown that the phagocytosis of *Mertk* knockout (*Mertk*^−/−^) macrophages was reduced and the cells failed to engulf apoptotic cells [70]. The MERTK-knockout-mediated inhibition of phagocytosis may be due to the disruption of cytoskeleton organization [154].

## 5. Development of Biological Agents and Small Molecules Targeting MERTK

TAM-mediated signaling pathways are responsible for the regulation of multiple biological processes, including survival, proliferation, cell mobility, and immunosuppression. The three members of the TAM family play important roles in the onset and progression of many diseases. Much evidence strongly suggests that these receptors play major roles in drug resistance to conventional chemotherapy and targeted therapies in NSCLC. Because MERTK and EGFR share similar downstream signaling pathways, targeting MERTK is a potential strategy for overcoming EGFR-TKI resistance in NSCLC.

The biological activities of MERTK can be mediated by its extracellular domain, which is required for the immune regulatory function and its intracellular domain, harboring kinase activity. Accordingly, anti-MERTK agents can be categorized as biological agents against the extracellular domain and small molecules targeting its intracellular kinase activity (Table 1). It has been shown that the treatment of a polyclonal anti-MERTK antibody (ab70693; Abcam, Cambridge, MA, USA) or homemade soluble MERTK ectodomain reduced the infection of classical swine fever virus and promoted the innate immune response [155]. A metastatic mouse model of lung adenocarcinoma showed that the combination of the anti-MERTK monoclonal antibody (4E9.E6; Bristol Myers Squibb, New York, NY) and anti-PD-1 monoclonal antibody (4H2; Bristol Myers Squibb) sensitized the tumors to radiotherapy by increasing the retention of CD8+CD103+ tissue-resident memory T cells in the tumors [156]. In addition, a preclinical study showed that Mer590, which is a monoclonal antibody, inhibits the MERTK-mediated signaling pathway, reduces colony formation, and sensitizes NSCLC cells to chemotherapy [95]. A homemade MERTK monoclonal antibody, which was derived by the extracellular domain of MERTK, significantly inhibited glioblastoma multiform migration [157]. In addition to the oncogenic roles of MERTK in cancer cells, MERTK expressed in stromal cells contributes to the immunosuppressive microenvironment. The inhibition of MERTK by the monoclonal antibody AF591 (R&D Systems, Inc., Minneapolis, MN, USA) blocks the apoptotic cell-induced inhibition of dendritic cells [158]. Interestingly, treatment of the MERTK monoclonal antibody has a limited effect on the bacteria-induced inflammatory response in macrophages, suggesting that MERTK plays a unique fundamental role in the tumor microenvironment, in addition to its role in systematic inflammation [159]. It is worth noting that although the three members of TAM receptors share the same downstream signaling pathways, the antibodies targeting MERTK, Axl, and Tyro3 may have different therapeutic efficacies or biological effects [160,161,162].

Interestingly, antibody-mediated MERTK activation has also recently been reported [160]. In contrast to Gas6 that can activate all three TAM receptors, the MERTK-activating antibody (AF591) and the Axl-activating antibody (AF854; R&D Systems) have absolute receptor specificity. An intravenous injection of the Axl-activating antibody strongly activates Axl receptors, leading to cleavage of the Axl extracellular domain from the cell membrane and the loss of steady-state Axl as a consequence in the spleen and lung, while the MERTK-activating antibody intensively activates the MERTK receptor, but is not associated with cleavage or loss of the MERTK protein, in the liver and lung of mice. However, the antibody alone is not able to induce apoptotic cell phagocytosis. In contrast, the antibody inhibits Gas6-mediated phagocytosis by competing with Gas6 for receptor binding [160]. In a mouse model of autoimmune encephalomyelitis, the Axl-activating antibody alone, but not the MERTK-activating antibody alone, reduced the number of inflammatory macrophages/microglia and the extent of demyelination, while the production of proinflammatory cytokines was not changed after the antibody treatment [161].

The upregulation of MERTK can be observed in recurrent tumors of cancer patients who received target therapy or chemotherapy as their first-line treatment. In colorectal cancer, the upregulation of MERTK has been considered as a predictive marker for resistance towards MEK1/2 inhibitors in a large clinical cohort [84]. The overexpression of MERTK was observed in melanoma cells with the BRAF V600E mutation, which are resistant to BRAF and MEK inhibitors [85]. In NSCLC, the excessive activation of Axl-mediated signaling pathways is associated with drug resistance to EGFR-targeted therapy [86,87,88]. A study using an in vitro cell culture showed that the overexpression of MERTK induced resistance to erlotinib in the PC9 cell line, which is an EGFR-TKI-sensitive cell line [89]. So far, clinical studies that form the basis of the different rationales for these combinations have been launched (Table 2). 

Due to the conserved sequences of the kinase domain, many compounds not only inhibit their primary RTK targets, but also exhibit biochemically inhibitory activity against TAM receptors. Several inhibitors were designed according to the molecular structure of MERTK, such as UNC569, UNC1062, UNC2025, and MRX-2843. Among these, UNC2025 induced the apoptosis of NSCLC cell lines carrying the EGFR T790M mutation, leading to EGFR-TKI resistance [93]. The application of the MERTK-specific inhibitor UNC569 sensitized EGFR-TKI-insensitive H1975 cells, which carry the EGFR T790M mutation, to the erlotinib treatment [93]. In addition, UNC1666 and UNC2250 are pyrrolopyrimidine- and pyridinepyrimidine-based MERTK inhibitors that inhibit the colony formation of NSCLC cells. Some other structurally-distinct MERTK inhibitors have been developed, such as CT413, RXDX-106, SGI-7079, UNC2541, and UNC3133. However, the therapeutic effects of these synthetic compounds have not been evaluated in a preclinical study of NSCLC.

The prominent expression and functions of TAM receptors across the immune system also suggest their potential role in mediating resistance to immune checkpoint inhibitors in cancer therapy. The activation of MERTK in macrophages promotes the phagocytosis of apoptotic tumor cells, which contributes to a macrophage-mediated immunosuppressive tumor environment [180]. The genetic depletion of MERTK or inhibition of MERTK by UNC2025 reduced the number of macrophages in tumors and sensitized the response of tumor cells to radiotherapy [156]. UNC4241and MRX-2843 alter the immune checkpoint pathway and increase T cell activation in a mouse model [181]. Furthermore, JNJ-28312141 and UNC2025 modulate inflammation and the transformation of macrophages [182]. Although the developed MERTK inhibitors display convincing therapeutic activities, there is no MERTK-targeting compound that has moved to a phase III clinical trial. In addition, the tumors of NSCLC exhibit heterogeneous expression patterns of MERTK [94]. Accordingly, the side effects of MERTK inhibitors in clinical application must be considered, due to the complicated crosstalk between MERTK and other RTK-mediated signaling pathways.

## 6. Conclusions

In the past decade, the contribution of the ligand-dependent canonical RTK-signaling pathway of MERTK in drug resistance has been extensively explored. Importantly, the activation of MERTK signaling pathways depends on the context in which the ligands and microenvironment are presented to the TAM extracellular domains. The complexity of ligand–receptor interactions reflects the difficulty of drug development for overcoming EGFR-TKI resistance in NSCLC. Recently, the combination of MERTK inhibitors and other therapeutic agents, such as immune checkpoint inhibitors, TKIs, and chemotherapy agents, has become the most promising strategy for fighting NSCLC. Although clinical studies have shown that anti-MERTK therapies potentially prolong the survival of NSCLC patients, the effectiveness of these compounds is not satisfactory. Future study may focus on the therapeutic efficacy among NSCLC patients with different genetic alterations of MERTK and with different EGFR-TKI-resistant mechanisms.

## Figures and Tables

**Table 1:** MERTK inhibitors in preclinical development for NSCLC.

Compounds	Target(s)	Experimental Models	Outcomes
Mer590 ^1^	MERTK/Tyro3	Cell culture	Reduction of MERTK protein levels, decrease in colony formation and chemosensitivity [95]
UNC1062	MERTK/Axl/Tyro3	Cell culture	Inhibition of A549 colony formation [163]
UNC2025	MERTK/Axl/Tyro3	Cell culture/ subcutaneous xenograft in mice	Inhibition of colony formation and tumor growth using multiple NSCLC cell lines [93]
UNC2250	MERTK/Axl/Tyro3	Cell culture	Inhibition of Colo699 colony formation [164]

^1^ Mer590 is a monoclonal antibody.

**Table 2:** The compounds harboring inhibitory activity of MERTK in clinical trials for NSCLC. ^1^ The inhibitory effect of individual compounds to corresponding primary targets has been approved by experimental data from biochemical, cell lines and animal studies. ^2^ The binding activities of compounds to their targets are screened by biochemical kinase selectivity assays. The inhibitory activities and cytotoxicity of some compounds are examined in cell lines. ^3^ Sources of information on clinical trials include ClinicalTrials.Gov (https://clinicaltrials.gov/ (accessed on 31 January 2021)), Canadian Cancer Trials Group Investigational New Drug (IND), International Standard Randomised Controlled Trial Number Register (ISRCTN) (https://www.isrctn.com/ (accessed on 31 January 2021)), and TrialTrove (https://citeline.com/ (accessed on 31 January 2021)).

Name	Primary Target(s) ^1^	Other Target(s) ^2^	Development Phase (Trials Registry Number ^3^)	Tumor Type ^1^
ASLAN002/BMS-777607	MET and RON	Axl, Flt3, MERTK and Tyro3	Phase I (NCT01721148)	Advanced or Metastatic Solid Tumours [165]
AT9283	Aurora kinases	GSK3β, FGFR2, JAK2/3, MERTK, RET, RSK2/3, VEGFR3 (Flt4), Tyk2 and Yes	Phase I (NCT00443976) Phase I (NCT00985868)	Advanced or metastatic solid tumors [166] Relapsed and refractory solid tumors
Bemcentinib/BGB324/R428	Axl	MERTK and Tyro3	Phase II (NCT03184571)	Advanced NSCLC
Bosutinib/SKI-606/PF-5208763	Src and Abl	MERTK, Axl, Tyro3, EphA2, Kit, Ste20, Tec and Trk	Phase I (NCT03023319) Phase I (NCT01001936) Phase II (NCT03297606)	NSCLC [167] Advanced malignant solid tumors Advanced solid tumors
Crizotinib/PF-2341066	ALK, MET and ROS1	MERTK, Axl, Tyro3, and RON	Multiple trials	MET+, ROS1+ or ALK+ advanced NSCLC
Dubermatinib/TP-0903	Axl	MERTK, Tyro3, AURKA, Flt3, AURKB, JAK2 and Abl	Phase I (NCT02729298)	EGFR+ NSCLC [168]
Foretinib/GSK1363089/XL880/EXEL-2880	MET and VEGFR2	MERTK, Axl, Tyro3, RON, PDGFR, KDR, Kit, Flt3 and TIE2	Phase II (NCT02034097) Phase I (NCT00742261) Phase I (NCT01068587) Phase II (IND.196)	NSCLC Solid Tumors Advanced or Metastatic NSCLC Advanced or Metastatic NSCLC [169]
INCB081776	Axl and MERTK		Phase I (NCT03522142)	Advanced solid tumors
Merestinib/LY2801653	MET	MERTK, Axl, Tyro3, MET, Flt3, RON, ROS1, Tek, DDR1/2, MKNK1/2	Phase I (NCT03027284) Phase II (NCT02920996) Phase I (NCT02745769)	Advanced or metastatic cancers [170] NSCLC Advanced cancers
Glesatinib/MGCD265	MET and VEGFR2	MERTK, Axl, Tyro3, Tek and RON	Phase II (NCT02544633) Phase I (NCT00975767)	NSCLC [171] Advanced NSCLC
MK2461	MET	MERTK, RON, Flt1, Flt3 and PDGFR	Phase I/II (NCT00496353) Phase I (NCT00518739)	Advanced solid tumors [172] Advanced cancers
MRX-2843	MERTK and Flt3	Axl and Tyro3	Phase I (NCT03510104)	Advanced and metastatic solid tumors [173]
Neratinib/HKI-272	HER2 and EGFR	MERTK, Axl and Tyro3	Phase II (NCT01827267) Phase II (NCT00266877)	HER2-activating mutations in NSCLC [174] Advanced NSCLC
ONO-7475	Axl and MERTK		Phase I (NCT03730337)	Advanced or metastatic solid tumors [175]
S49076	MET	Axl, MERTK and FGFR	Phase I (ISRCTN00759419)	NSCLC [176]
Sitravatinib/MGCD516	spectrum-selective RTKs	MERTK, Axl, Tyro3, VEGFR2, PDGFR, Kit and MET	Phase I (NCT02219711) Phase III (NCT03906071) Phase II (NCT02954991) Phase I (NCT03666143) Phase II (NCT02664935)	Advanced cancers [177] Metastatic non-squamous NSCLC NSCLC Advanced solid tumors. NSCLC
SU-14813	multi-targeted RTKs	MERTK, Axl, Tyro3, FLT3, VEGFR, PDGFR, Kit	Phase I (NCT00982267)	Advanced solid tumors [178]
Vandetanib/ZD6474	multi-targeted RTKs	MERTK, Axl, Tyro3, VEGFR2, VEGFR3, EGFR and RET	Multiple trials	NSCLC [179]

Abbreviation: MET, MNNG HOS transforming gene; RON, recepteur d’origine nantais receptor; Flt3, Fms-like tyrosine kinase 3; GSK3β, glycogen synthase kinase 3 beta; JAK, Janus kinase; RSK, ribosomal s6 kinase; AURKA, Aurora Kinase A; AURKB, Aurora Kinase B; RET, rearranged during transfection; VEGFR, vascular endothelial growth factor receptor; Tyk2, tyrosine Kinase 2; Yes, tyrosine-protein kinase Yes; Src, v-src avian sarcoma (Schmidt-Ruppin A-2) viral oncogene homolog; Abl, v-abl Abelson murine leukemia viral oncogene homolog; EphA2, erythropoietin-producing hepatoma (Eph) receptor tyrosine kinase A2; Ste20, sterile 20 protein kinase; Tec, Tec Protein Tyrosine Kinase; Trk, tropomyosin receptor kinase; ROS1, c-ros oncogene 1; ALK, anaplastic lymphoma kinase; VEGFR2, vascular endothelial growth factor receptor 2; KDR, kinase insert domain receptor; Tie2, tunica interna endothelial cell kinase 2; DDR, discoidin domain receptor; Tek, TEK receptor tyrosine kinase; MKNK1/2, MAP kinase-interacting serine/threonine-protein kinase 1/2; PDGFR, platelet-derived growth factor receptor; Kit, v-kit Hardy-Zuckerman 4 feline sarcoma viral oncogene homolog; and FGFR, fibroblast growth factor receptor.
